# Infant formula feeding practices and the role of advice and support: an exploratory qualitative study

**DOI:** 10.1186/s12887-017-0977-7

**Published:** 2018-01-24

**Authors:** Jessica Appleton, Rachel Laws, Catherine Georgina Russell, Cathrine Fowler, Karen J. Campbell, Elizabeth Denney-Wilson

**Affiliations:** 1 Faculty of Health, University of Technology Sydney, Broadway, P.O. Box 123, Sydney, NSW 2007 Australia; 2 Sydney Children’s Hospital Network, Sydney, Australia; 3 Deakin University, Institute for Physical Activity and Nutrition, Locked Bag 20001, Geelong, VIC 3220 Australia; 4 Centre for Obesity Management and Prevention Research Excellence in Primary Health Care (COMPaRE-PHC), Sydney, Australia; 5 Tresillian Chair in Child and Family Health, Faculty of Health, University of Technology Sydney, Sydney, Australia; 6 Tresillian Family Care Centres, Belmore, Sydney, NSW 2192 Australia

**Keywords:** Infant formula, Obesity, Overweight, Parents, Mothers, Feeding behaviour, Marketing

## Abstract

**Background:**

Infant formula feeding practices are an important consideration for obesity prevention. An infant’s diet is influential on their later risk of developing overweight or obesity, yet very little is known about infant formula feeding practices. It is plausible that certain modifiable practices may put children at higher risk of developing overweight or obesity, for example how much and how often a baby is fed. Understanding how parents use infant formula and what factors may influence this practice is therefore important. Moreover, parents who feed their infants formula have identified a lack of support and access to resources to guide them. Therefore this study aimed to explore parents’ infant formula feeding practices to understand how parents use infant formula and what factors may influence this practice.

**Methods:**

Using an explorative qualitative design, data were collected using semi-structured telephone interviews and analysed using a pragmatic inductive approach to thematic analysis.

**Results:**

A total of 24 mothers from across Australia were interviewed. Mothers are influenced by a number of factors in relation to their infant formula feeding practice. These factors include information on the formula tin and marketing from formula manufacturers, particularly in relation to choosing the type of formula. Their formula feeding practices are also influenced by their interpretation of infant cues, and the amount of formula in the bottle. Many mothers would like more information to aid their practices but barriers exist to accessing health professional advice and support, so mothers may rely on informal sources. Some women reported that the social environment surrounding infant feeding wherein breastfeeding is promoted as the best option leads a feeling of stigma when formula feeding.

**Conclusions:**

Additional support for parents’ feeding their infants with formula is necessary. Health professionals and policy around infant formula use should include how formula information may be provided to parents who use formula in ways that do not undermine breastfeeding promotion. Further observational research should seek to understand the interaction between advice, interpretation of cues and the amount formula fed to infants.

**Electronic supplementary material:**

The online version of this article (10.1186/s12887-017-0977-7) contains supplementary material, which is available to authorized users.

## Background

How and what an infant is fed during the first year of life is fundamentally important to the prevention of childhood obesity [1]. Whether an infant is breastfed, formula fed or mixed fed with both breast milk and infant formula (herein called formula) may affect their risk of developing obesity later in life. Many studies have found breastfeeding can reduce the risk of developing obesity later in life [2–5]. However, evidence for this association remains equivocal. This may reflect study definitions and design – for example some studies have not addressed important confounding variables or have used varied definitions of the duration or exclusivity of breastfeeding. It may be that the impact of breastfeeding on weight is less obvious when infants mix fed with breast and formula milk are classified as ‘breastfed’. Another consideration is how the type of milk feeding may influence infant weight gain. Infants who experience excess or rapid weight gain in infancy are more likely to be overweight or obese in childhood [6, 7].

The mechanisms underlying the associations between the type of milk feeding and risk of developing obesity later in life are not well understood, however, there are a number of theoretical pathways that may explain this relationship. For example, a recent study has shown a positive relationship between higher protein content formula and excess weight gain in infancy [8] and obesity in childhood [9]. Another study found an association between use of a commercial milk cereal drink at six months and higher body mass index at 12 and 18 months [10]. Additionally, the domain of responsive feeding, (that is the relationship between an infant’s cues of hunger and satiety and a parent’s perception and response to these cues), are important considerations in obesity prevention [11, 12]. Together these factors have implications for formula feeding practices which include the type of formula, the preparation of formula, the amount provided and consumed, and the way in which formula is fed, for example feeding to schedule or demand.

Despite the emerging evidence suggesting that there are modifiable formula feeding practices that may contribute to the excess weight promoting effects of formula feeding very little is known about how parents use formula to feed their infants. We know generally that parents use both formal and informal sources of information and advice to guide them in how and what to feed their infants [13] and it appears a number of factors, such as everyday situations like a holiday or illness, and previous experience also influence these practices [14]. Yet, we know little about which specific resources are accessed when making formula feeding decisions. This is important because information, advice and support for parents using formula has been found to be inadequate or missing completely [15–17]. Recent research has found that parents feeding with formula have felt unsupported by health professionals that are meant to help and support during infant rearing, such as the midwives and maternal and child health nurses [16–18]. The importance of supporting parents with high quality advice and support is underscored by the findings from a number of intervention studies. For example interventions that include professional support to promote breastfeeding for parents have been found to succeed in increasing the initiation and duration of breastfeeding [19].

Formula feeding practices, and infant feeding in general, occurs within a family and a cultural society with expected norms and values [20]. In many countries, including Australia, the expected norm is to breastfeed [21]. This focus on breastfeeding has meant formula feeding is often viewed as the ‘second best’ option [22]. On the other hand, within certain demographic and cultural groups, breastfeeding may not be the expected norm [23]. Nevertheless, some parents using formula have reported feeling judged because of their choice to use formula [24]. These values and norms can influence the type of advice parents receive [23].

Considering there are formula feeding practices, for example feeding according to infant hunger and satiety cues, that may reduce the risk of excess or rapid weight gain it is crucial to understand how parents use formula to feed their infants and what factors influence their formula feeding practices. This qualitative study aims to explore parents’ formula feeding practices and the factors influencing this practice, as well as exploring the source of advice used by parents.

## Methods

### Study design and participants

This study utilised a pragmatic qualitative inquiry design [25] with thematic analysis informed by Ritchie and Lewis’s [26] stage approach. This study recruited parents from an Australian longitudinal cohort study, Baby’s First Foods (BFF). Parents were initially recruited into BFF when their infant was aged three months or younger through advertising on websites, online parenting forums and Facebook pages from February–April 2015. Parents were eligible to participate in BFF study if they were 18 years or older, they were literate in English, and were living in Australia. Parents completed online surveys at recruitment and when their infant was around six and around nine months of age. At the nine month survey participants were asked if they would participate in a telephone interview exploring their experience of feeding their baby. After the final survey, parents who had agreed to participate and had used formula during the first nine months were invited via email and a follow up call, and a telephone interview time was arranged. Of the eligible parents, participants were purposefully sampled so that the interviewees represented parents with different levels of education, with first born or subsequent children, mixed or exclusive formula feeding and age of infant when starting formula. Participants received a $30AUD gift card to compensate them for their time. Ethics approval for this research was granted by the University of Technology Sydney and the Deakin University Human Research Ethics Committees.

### Data collection

Semi-structured interviews were used to explore parents’ formula feeding practices, the factors that influenced these practices and their experiences of seeking or being provided with information, advice or support for formula feeding [see additional file 1 for interview guide]. Data were collected between November 2015 and February 2016. Telephone interviews lasting on average 35 min were digitally recorded and transcribed verbatim. Interviews were conducted by the lead author and transcripts were checked by the interviewer against the recordings. All identifying information was removed during transcription checking and confidentially of the participants and their infant has been kept though use of a pseudonym and replacing the infants’ names in the transcripts with ‘baby’.

### Data analysis

A pragmatic inductive approach to analysis was used, informed by Ritchie and Lewis’s [26] stage approach. This involved familiarisation with the dataset through reading the transcriptions and listening to the audio recording [26]. Initial codes were then generated. These remained close to participants’ own language and understanding, creating a thematic coding framework informed by the interview questions and codes identified. The data were then coded by this framework, which remained open to additional codes, refining and clustering of codes [26]. Finally, this involved establishing typologies, detecting patterns and explanatory accounts of these patterns [26]. This was an iterative process involving, at first, descriptive accounts of the data through to interpretive accounts, which conceptualised the final themes [26]. All coding and analysis was conducted by the first author. To address rigour, an audit trail was kept including documentation of the analysis process, lists and structure of the thematic framework and codes. Regular coding meetings were held between the first author (JA), and two other authors (RL and EDW) to discuss the thematic code template and provide additional insights. Data were managed using Nvivo data management software [27]. In a similar manner to previous studies [16], the participants were classified into groups according to their feeding history (i.e. formula fed: an infant was exclusively formula fed within their first six weeks; switched early: breastfeeding initiated but exclusive formula feeding commenced within the first six months; switched late: initially breastfed but formula feeding commenced after six months or mixed fed with both breast milk and formula).

## Results

### Participant characteristics

Of the 51 participants contacted, 25 agreed to participate and were interviewed. One participant’s interview was removed from analysis as the infant was admitted to the special care nursery at birth which influenced the way that infant was fed. Overall interviews from 24 mothers of infants aged between nine and 11 months were analysed. This sample had varying experience with formula, with some participants starting to feed with formula at eight months, others ‘mixed feeding’ (i.e. breastfeeding alongside formula feeding) from birth to the time of the interview, and others feeding with formula exclusively from the first few weeks. Table 1 shows sample characteristics.

**Table 1:** Participant Characteristics

Characteristics	*n* = 24
Age of mothers (years)	Range 21–39
20–24	3
25–29	8
30–34	7
35–39	6
Age of infant (months)	Range 9–11 months
9	1
10	15
11	8
Infant gender	
Male	7
Female	17
Number of children	
1	10
2+	14
Mother’s education	
Tertiary (university) or higher	11
Trade or diploma	8
High school	5
Location – state	
NSW	5
NT	1
QLD	5
VIC	10
ACT	3
Location – Remoteness^a^	
Major City (RA1)	13
Inner Regional (RA2)	9
Outer Regional (RA3)	2
SEIFA – decile^b^	
< 2	2
3–4	7
5–6	2
7–8	6
> 9	6
Infant feeding	
Formula fed^c^	6
Switched early^d^	7
Switched late^e^	5
Mixed fed^f^	6

^a^Based on the Australian Standard Geographical Classification – Remoteness Area (2006) which classifies from RA1 Major City to RA5 Very Remote [50, 51]

^b^Socio-economic Indexes for Areas 2011 by post code, the decile is the rank order of all areas across Australia -this is a measures are a calculation of the location and not necessarily indicative of individuals in the location [52]

^c^Infant was fully formula fed within first 6 weeks

^d^Infant commenced formula between 6 weeks - 6 months and was fully formula fed by 6 months

^e^Infant commenced formula after 6 months

^f^Mixed fed between birth and 6 months (any duration) and continued mixed feeding or moved to full formula feeding after 6 months

Analysis of the transcripts identified six themes, with one theme having four subthemes. Three of the themes concerned factors that influence the how and why of parent’s formula feeding practices, these were titled: ‘Choice of formula – what’s on (and in) the tin’; ‘Bottle preparation - mostly by the tin’; and ‘How much and how often’ which had four subthemes. The next two themes concerned sources of information, advice and support about formula, these were titled: ‘informal advice’; and ‘formal advice’. The final theme entitled ‘Bottle stigma’ concerned the social environment surrounding infant feeding practice.

### The how and why of parents’ formula feeding practices

Parents described how they selected a formula brand and type, how they prepared a bottle of formula and how they determined how often and how much milk to offer at each feed. Parents used the information on the tin to choose the brand, and for advice about how to prepare the formula, how much to offer each feed and in some cases the pattern of feeding. An additional file provides further samples of supporting quotes for these themes [see Additional file 2].

#### Choice of formula – what’s on (and in) the tin

Most parents commented that their choice to remain with a formula was influenced by their assessment of the suitably for their infant. One participant explains *…but ultimately does the baby drink the formula? Are they settled? If not then try a different formula* (Charlotte, switched late). However, a combination of factors influenced the initial choice of formula, such as whether it was made in Australia, or that it was labelled organic: *I think just that… it was organic and it was Australian owned company …I felt comfortable with that brand* (Maya, switched early).

Other factors such as previous experience, availability of a brand, advice from other parents and health professional recommendations were cited by some respondents. For other parents identifying a specific type of formula such as ‘hypoallergenic’ was important. Some respondents indicated that marketing attributes such as community trust of the brand, if the brand provides single serve sachets, and advertising were factors in their decision making:

*But I think it all comes down to the advertisement on the tin. That’s what you’re reading. I know you get warned so many times that advertisement on foods half the time they’re not really true but what have we got to go by? The health care professionals they’re not telling us which one’s the best one to go for.* (Charlotte, switched late).

The parents also explained that they considered other sections of information on the tin such as the list of ingredients or nutrition panel, but others expressed confusion about whether this information could be trusted and who could help them in understanding. While some parents did received guidance from health professionals, many noted there was little information provided by health professionals (or elsewhere) to aid their decision.

#### Bottle preparation - mostly by the tin

Parents described preparing the bottle of formula according to the instruction on the formula tin. It is usual for formula tins to have instructions with picture aids about how to prepare a bottle including sterilising, using cooled boiled water, putting the water in first (then adding the powder) and using the enclosed scoop to accurately prepare the correct concentration. Most parents reported that they followed these instructions; one participant stated *I’m pretty bang on with the powder that I use. I think the proportions are pretty important* (Imogen, switched late). For some parents they occasionally deviated from the instructions, for example using a microwave, or not sterilising the bottle. Two parents also noted that they added a little extra water under health professional advice: *At one point the paediatrician told me to add 20 ml extra of water to each bottle to help with constipation…* (Layla, switched early).

#### How much and how often

Parents were influenced by a number of factors in deciding how much and how often to feed. These factors have been categorised into four subthemes: ‘demand versus routine’ – whether to feed to demand or feeding to a routine; ‘balancing responding to baby’s cues (of hunger and satiety) versus the information on the tin’ (recommending how much an infant should have); ‘parents’ perceptions of other external cues’, such as how much milk was in the bottle, the time, or how much milk the infant consumed at a previous feed; and ‘getting advice’ - seeking and receiving advice regarding frequency and volume of feeds.

##### Demand versus routine

Many parents spoke about using both demand and routine feeding. Often initially, when the infant was younger they used demand feeding, where they would feed according to their interpretation of their infant’s cues. However, many described that out of this an organic routine emerged:

*I demand feeded [*sic*] in the beginning. Because he had been breastfeed before that, I kind of knew his roughly when he would feed. Formula sort of spaced that out a little bit.* (Lucy, formula fed).

A few used a specific feeding regime, for example this participant explains *I already had a routine set with her …so pretty much we stuck to the every three hours, so six, nine, 12* (Ellie, switched early). Other factors of the daily routine influenced when an infant was fed such as infant sleep habits or mother routine, and for some it was based on the time since the last feed.

##### Balancing responding to baby’s cues versus the information on the tin

How much and how frequently to feed was influenced by a combination of parents’ interpretation of their baby’s cues and reading the advice on the formula tin. Overall, parents explained they tried to read and follow their infant’s cues. Parents’ descriptions of their infant’s cues when their infant was younger and older were different, with older infant’s cues being more overt, for example:

*So she kind of changed as she got older …when she was younger it was more that she had enough, she was just more interested in the dummy like playing around with the teat rather than actually drinking it. And, as she got older she kind of just like shoved her head out of the way and just pushed the bottle away and was like “no I’m done”…* (Kim, formula fed).

This process of reading and interpretation of infant cues was also often described as a learnt process and a matter of ‘trial and error’.

 Yet this learnt process was also influenced by the advice provided on the formula tin. Formula tins provide suggested volumes and feeds per day and most participants, to a greater or lesser extent, used this as a guide. Two parents also said it influenced the timings of each feed. About half the parents saw this as a flexible guide, and they adjusted according to their infant’s consumption. For most others they continued to stick strictly to the amount the tin recommended even though their infant often did not drink that amount. For two parents, the recommendation was described as a firm guide. For example one parent described increasing the amount of formula as per the tin recommendations until the infant consumed the amount recommended for their age:

*If she wasn’t interested, if she was a lot behind what the tin was telling me… put it back in her mouth and she started crying, it meant she just was not interested at all so I would tip out the 10 ml or however much she left but I’d continue to make the bottle up to that volume until she then took it on* (Alyssa, switched early).

##### Perceptions of other external cues

In addition to the tin advice, other external factors were also considered as cues for when and how much to feed. Some parents perceived the amount of milk in the bottle an indicator or cue that helped them interpret their infant’s appetite. These parents explained that they would offer the bottle again to check, or ensure that their infant had had enough milk. One mother explains, …*if he spat out the bottle I would pretty much, sit him up burp him and try the bottle again and if he pushed it away a second time then I knew he was full* (Ruby, switched late). Other parents’ descriptions showed that finishing a bottle was an indicator to them of their infant’s appetite, for example:

*I don’t want to start giving her less than 180 because more often than not now she finishes the bottle. That tells me then that probably she either needs it or she wants it* (Chloe, switched late).

If the infant finished the amount of milk in the bottle this showed their parent that they had had enough milk. Some parents then also based the amount of milk they made up on the amount the infant had previously consumed. Once the infant began eating solid foods, the parent’s perception of how much solid and semi solid foods they had eaten also influenced how much formula the infant needed:

*I might see how she goes with 210, so that’s usually at night if she hasn’t had much dinner or hasn’t looked like she’s hungry or if I’m concerned about what she’s had during the day, I might make up the 210 to see how she goes* (Chloe, switched late).

##### Getting advice

Lastly, external sources of advice such as health professionals, family or other mothers also influenced how much and how often parents fed their infant. For some this advice was given unbidden, and for others they sought advice for a specific query they had. For example, some parents were unsure if it was okay for the infant to drink less than the recommended amount on the formula tin, and this was resolved through reassurance from other mothers or discussions with health professionals.

A few parents were advised by a health professional to calculate the volume of each feed and the total volume in 24 h based on an equation that takes into account their infant’s weight. Although this equation was often provided by a health professional, one mother found it on the internet: *I Googled and I found everywhere that it was 150 mls by their weight or something. So that’s what I did…* (Zara, formula fed).

### Sources of information, advice and support about formula and social environment

Participants reported using informal and formal sources of advice about formula feeding. Informal sources included the tin, the internet, family, friends and other mothers. Parents use of these is described in the theme "Informal sources of advice". Formal sources includes midwives and doctors at the birth hospital, community maternal and child health nurses, general practitioners, paediatricians and pharmacists. However, a number of parents would have liked more information particularly from health professionals and reported challenges to receiving advice from formal sources- described in the theme ‘formal sources of advice’. The final theme ‘Bottle stigma’ will address parents’ experience of the social environment surrounding infant feeding practice. In addition to the in text supporting quotes an additional file provides further samples of supporting quotes for these themes [see Additional file 3].

#### Informal sources of advice

Mothers used the tin, family or friends, other mothers and the internet (including social media and commercial websites) to gain advice about formula:

*…so looking on the tin, looking online and like family members that have already gone through that experience because they might have fed formula to their kids. The other I suppose information source would be my mother’s group as well...* (Savannah, mixed fed).

While many parents did use some formal professional sources of information the majority said that informal sources had an influence on what they did and was often the place they would turn to when they had questions, one participant explains *I would say friends and family, I think they’re probably the biggest influence* (Amelia, formula fed). It was their local support network of family, friends and other mothers who were key resources that influenced their formula feeding practices and feeling of support when formula feeding. The internet was also another place some parents found information:

*Yeah that’s where I got a lot of my information, from the internet. Not all websites are good and a lot of it was contradicting other information. But I found the internet my best source of information definitely* (Zara, formula fed).

Some of the parents were satisfied with the resources available to them, that they were sufficient for their needs. They did not feel they had a need for any other resources. This may have been influenced by the perceived ease of using formula and that they had minimal or no specific problems for which they needed advice, as one participant explains *Well I just mainly just follow the packet instructions. I didn’t really think about it too much, just following the packet and went with it and fed her when she was hungry* (Kim, formula fed). Using formula was perceived as easy for these mothers, particular compared to other infant feeding areas such as breastfeeding or starting solids.

#### Formal sources of advice

A number of mothers felt they would have liked more information, particularly from health professionals:

*…it would have been very helpful to have professionals have some sort of information on hand when they find out that you are formula feeding to make sure that you are making it up correctly, giving the right amount and what types of formula are out there that are beneficial* (Ellie, switched early).

For mothers who did receive information it was often after starting formula. For example:

*[Did you get any information about formula before you started using it?] Only, other than - My sister has a baby, so she’s 18 months older than [baby]. Other than just conversations that I had with my sister and conversations that I’ve had with my mum, not really* (Chloe, switched late).

Although this may have been influenced by the swiftness needed in making the decision to start or change the type or brand of formula:

*It was open late night and I was desperate, I thought I needed to put her on something. So I went to the chemist… the [brand], they have individual sachets… and that’s what I bought for the first time, just to see if she was going to take formula or not* (Amelia, formula fed).

Additionally, many parents had negative experiences with some health professionals, which led to perceived barriers to access their advice and support for parents. One of these barriers was that parents found health professionals didn’t actually talk to them about formula feeding unless they specifically asked, this participant explains *It’s almost like a taboo subject at that very moment so in hospital and at that point I really would have liked information* (Emma, switched early). Another barrier was they felt health professionals pushed ‘the breastfeeding’ line and that they were judged negatively because they were formula feeding:

*From the MAC [maternal and child health] nurses, very judgemental. They do push ‘breast is best’ and to the point of making you feel really bad* [later this participant notes] *…I talked to the MAC nurses they were too judgemental. So that’s why I’ve never really visited them this time around* (Layla, switched early).

On the other hand there were positive experiences where parents felt comfortable going to health professionals; although often the parent had to be proactive in seeking this advice or support. For a few parents their negative experience with one health professional was followed by a positive experience with another who was sympathetic to their situations and did support them:

*…when I had made a choice that the very first person that I’d spoken to wasn’t – like it didn’t feel like they were supporting me in my choice…But I then found other people in the medical industry who were supportive of me and yeah I’m glad that I did find them* (Ruby, switched late).

#### Bottle stigma

Parent’s sense that professionals prefer breastfeeding carried over to everyday life. Some, but not all, parents felt that they were judged not just by health professionals but in their community and general society too. This led to feeling that using formula has a stigma attached to it, and parents sometimes felt judged particularly when they first started using formula. One participant shares, *I think there’s a lot of stigma attached to formula use… So if you’re out there’s a lot of scrutiny if you pull a bottle out* versus *if you breastfeed your child* (Evelyn, mixed fed).

On the other hand, a small number of parents actually felt they were more supported using formula, and/or wanted more support in their breastfeeding. These parents felt their family and immediate social support had an influence on their success in breastfeeding as these support structures encouraged using formula, for example this participant reflects that her family influenced her *I had family members telling me to give her formula and all that…* (Isla, switched early). It is interesting to note that the four participants who identified this factor were all mothers under 30 years of age.

## Discussion

This study provides insights into the use of formula and mothers’ experience of advice and support for their formula feeding practices.

Feeding in response to cues of hunger and satiety has become an important focus in obesity prevention in infancy and childhood [28]. Previous work in the United Kingdom developing a questionnaire of maternal attitudes towards infant growth and milk feeding practices found the tin, growth and appetite as potential factors used by parents to identify how much formula to offer [29]. However, how external cues, such as the advice on the tin or the amount of milk left in the bottle, influence parents’ perception of an infant’s appetite is unclear. The current study found that for some parents there were interactions between interpreting their infant’s cues and other factors, such as the time between feeds, the amount of milk in the bottle, or the infant finishing the bottle. This could potentially lead to the infant receiving more formula than they need. A recent laboratory based study to test the impact of the bottle as a cue for mothers found that mothers with a pressure feeding style were more responsive to their infant’s cues when feeding with a weighted opaque bottle compared when feeding with a conventional clear bottle [30] suggesting that some parents may use bottle fullness to inform their view of their child’s satiety.

Of the few studies that have assessed how much formula infants are consuming, findings reveal that infants fed formula often consume more milk than breastfed infants and current recommendations. For example, in a study of 43 infants during the first two days of life infants fed with formula consumed significantly higher amounts of milk (over double the amount) than breastfed infants [31]. In another more recent study fully formula fed six week old infants (*n* = 319) consumed a mean of 205 ml/kg body weight/day [32], compared to current recommendation of 150 ml/kg body weight/day. Considering these findings, parents use of ‘the bottle’ and other external cues to interpret their infant’s appetite, how this related to the infant’s cues of hunger and satiety, and if this is linked to formula consumption warrants further research.

Understanding the factors that impact on infant expression of cues and parent interpretation of cues is important [33], particularly in formula fed infants as recent findings suggest they display fewer engagement (hunger) cues and disengagement (satiety) cues over the duration of a milk feed than breastfed infants [12]. Previous research has also found variation in parents’ explanations of their infant’s hunger and satiety cues across the first year of life [11]. Similarly, the current study found variation in the way parents described their infant’s hunger and satiety cues across time, with cues becoming clearer to parents as the infant aged.

Another important finding of this study is the potential impact formula manufacturers’ marketing may have on how parents use formula, particularly the brand and type they choose to use, and the way formula is made up and the amount provided. The information on the tin including marketing factors such as health claims was described as informative and influenced the choice of formula for around half of the parents in this study. Parents based their decision on what they thought was most important, yet they were unsure how to interpret the information on the tin and which pieces of information they could trust. It seems that the images and text on the tin are influential pieces of marketing. It is noteworthy that parents were exposed to formula advertising considering the current restriction of advertising formula for infants under one year old in Australia under the Marketing in Australia of Infant Formulas (MAIF) Agreement [34]. Recent research in Australia and Italy has shown that parents are likely to interpret toddler formula advertising as infant formula advertising [15, 35] and this is concerning because toddler formula advertising is not subject to the restrictions in place for infant formula advertising across a number of countries including the United States, Canada, the United Kingdom (UK) and Australia [36].

Considering this environment, it is important that non-commercial sources of advice and support are available to parents using formula [37], specifically, advice that would help parents to interpret infant formula marketing and make an informed choice, rather than relying on their own perceptions of quality (for example, Australian made or organic). This is particularly important as there is an increasingly diverse range of formula from which to choose and there may be important differences between formula brands. For example, within the formulas found on the Australian market the protein content ranges widely from 13 g/L to 19 g/L in formulas design to be used from birth [38]. This is particularly important for obesity prevention as recent research has found formula with a low protein content may reduce excess weight gain in infancy [8] and reduce risk of childhood obesity [9]. Yet, as the current study shows that many parents felt there is not information or advice to help guide them.

This current study, consistent with many recent studies, has found anticipatory and ongoing guidance or advice from health professional sources for formula feeding is perceived by parents as both necessary and deficient [17, 18, 32]. A study based in the United Kingdom also found both community and hospital based midwives were limited in their knowledge of infant formulas and these midwives acknowledged that those parents that formula feed receive less information and support than those who breastfeed [39]. Another recent assessment of infant feeding support services in regional New South Wales, Australia, found services such as written or verbal education, and support for formula feeding were inadequate [40]. The current study found that many parents did not discuss using formula with a health professional before they started feeding with formula. This is interesting as formula tins sold in Australia carry a label in accordance with the International Code of marketing of breast-milk substitutes [34, 41], advising that breastfeeding is the optimal infant nutrition and that parents should seek health professional advice before using formula.

Barriers to parents consulting with health professionals highlighted in this study included the perceived haste with which formula is commenced, the perception that health professionals do not endorse formula and the easy access to other avenues of advice and support such as commercially provided information on the tin, friends, family and the internet. That parents in the current study used non-formal sources of advice regarding formula use is consistent with other studies [13, 32]. Interestingly, in a study based in Ireland, those parents who formula fed their infant from birth tended to use more informal sources of infant feeding information than those who mixed fed [32]. The extent to which informal information, such as from the tin and the internet influence formula feeding practice warrants further investigation.

The current discourse and practice around infant feeding guidance has a large focus on breastfeeding. Results of the current study found health professionals do not talk about formula and they pushed the ‘breastfeeding line - breast is best’. Mothers in a Scottish study exploring their postnatal experience of infant feeding, found there was a ‘perceived reluctance’ of health professionals to provide parents with information about using formula [16]. A study of Australian antenatal classes found that health professionals focussed on breastfeeding and sometimes portrayed formula in a negative light and as potentially harmful [42]. Additional qualitative studies with mothers making infant feeding decisions show mothers felt some pressure to breastfeed from the health systems [18, 43]. While clearly it is vital that health professionals do support breastfeeding, in line with current global health strategy, policy and evidence base [44, 45], this focus may result in parents not approaching health professionals while considering formula use and not seeking support while using formula. Recent research in this area has called for feeding support that is more individual to feeding style, empathetic to parents’ choices [46], family centred [47] and specifically provides support for formula feeding so parents do not have to rely on commercial information [37].

The public health message of breastfeeding promotion not only influences the interaction between parents and health professionals but permeates through many sociocultural environments and into women’s self-perception of what it means to be a ‘good’ mother [43, 48]. Failure to breastfeed or breastfeed for the duration they intended, can cause many negative emotions including feelings of guilt [17]. The current study found that negative community perceptions of formula feeding are palpable to parents. In line with other recent research there was a sense of ‘bottle stigma’ and guilt related to using formula, which may have implications for perinatal mental health [46]. Additionally, while breastfeeding is considered the norm in many communities, there continue to be areas where formula feeding is most common, perceived as normal and where those choosing to breastfeed may lack support with implications for the duration of breastfeeding.

### Study strengths, limitations and further research

Strengths of this study include the recruitment of a mothers from across Australia which provides rich, varied viewpoints. The sample also included various infant feeding methods and lengths of breast, formula and mixed feeding allowing for a range of experiences to be explored.

A potential limitation of this study was that the interviews were conducted via telephone. Telephone interviewing removes physical cues of communication present in face to face interviews and may limit the depth of information exchanged [49]. However, there are also strengths to the use of this methodology given telephone interviews may promote sense of anonymity which may in turn promote openness in expressing views [49]. The use of telephone interviews also enables mothers to remain in their own environment which can make them feel more comfortable, along with offering greater flexibility in interview times and the ability to include participants not located close to the researcher [49]. Further limitations of this study include the interviews provide only the mother’s perspective (as no fathers were interviewed) and the potential for recall bias as the interviews were conducted when the infant was aged between nine and 11 months. A final potential limitation is that mothers’ described their formula feeding practice, rather than this practice being observed.

This study has begun to address a gap in our current understanding of how formula is provided on a day to day basis to infants and if potentially weight promoting infant feeding practice are common. However, further observational studies in this area may find different results, particularly of caregiver interpretation of cues and how this influences formula feeding practice. Additionally, further research to identify what sort of support parents using formula need and any barriers to accessing support for parents using formula to feed their infant is warranted. In addition research to understand health professional practices and experience of providing information and advice about formula to parents, and if there are barriers to provided information or advice about formula and what these barriers may be.

## Conclusion

Formula feeding practice is influenced by a number of factors, including the infant’s cues of hunger and satiety, other external cues such as the amount of milk in the bottle, and external sources of advice such as that provided on the infant formula tin and other forms of marketing. The current public health and health professional messaging regarding the avoidance of infant formula creates an environment where some mothers may feel unsupported thus discouraging parent’s access to health professional advice or support. In turn these mothers may seek information regarding this important period of infant feeding from informal sources such as family, friends, the internet, or commercially provided information.

## Additional files

Additional file 1: Semi-structured interview guide. (DOCX 18 kb)

Additional file 2: The how and why of parents’ formula feeding practices – further supporting quotes. (DOCX 21 kb)

Additional file 3: Sources of information, advice and support about formula and social environment – further supporting quotes. (DOCX 19 kb)

## Abbreviations

(BFF): Baby’s First Foods study
(MAC): Maternal and child health nurse
