# Supplementary material for: Infant formula feeding practices and the role of advice and support: an exploratory qualitative study
Source: BMC Pediatr. 2018 Jan 24;18:12. doi: 10.1186/s12887-017-0977-7 (PMC5784678; doi:10.1186/s12887-017-0977-7)
Supplement: Supplementary file 1 — Semi-structured interview guide. (DOCX 18 kb) [file 12887_2017_977_MOESM1_ESM.docx]

**Additional file 1 Semi-structured interview schedule**

| Questions about specific formula feeding practices and how the parent decides upon which formula feeding practices to use |
| --- |
| Thinking back to when you first started using formula   1. How did you decide which brand of formula to buy? 2. Where did you buy the formula from? 3. How did you decide when in the day to feed your baby? 4. When you were feeding your baby, how did you know when to stop feeding? 5. When you made up a bottle - how did you know how much formula to make up? 6. When thinking about making up bottles was there a time when you did not followed what it says on the tin?   Now that your baby is older   1. Do you still use the same formula? 2. How do you decide when in the day to feed your baby? 3. When you are feeding your baby, how do you know when to stop feeding? 4. When you made up a bottle - how do you know how much formula to make up? |
| Questions about experiences of seeking information, advice and support, and how this influenced their formula feeding practices. |
| 1. Did you receive any information about formula before you decided to use formula? 2. Would you have liked to receive information during the antenatal and early postnatal period about formulas? 3. At the time you started using infant formula did you received any information, advice and support from a health professional? (e.g. GP, nurse or midwife) 4. What is your experience of seeking information, advice and support about infant formula? 5. When you needed to, where or whom did you turn to for information, advice and support about formula? 6. Was there anywhere else that you found information, advice or support to help you in using formula? (explored if they used a number of sources that may not have come up in the interview so far e.g. pharmacist, practice nurse, websites) 7. From all the different sources of information, advice and support, who or what influences/influenced what you do? 8. What sources of information have you found helpful in specific areas, such as:    1. The brand and type    2. How much to make up per feed    3. When to feed during the day    4. Making up a bottle of formula 9. Are there any areas where you would have liked more information or support on with regards to feeding? 10. In general, have you felt supported in your infant feeding? If so, who did you feel supported by? |
