# Supplementary material for: Infant formula feeding practices and the role of advice and support: an exploratory qualitative study
Source: BMC Pediatr. 2018 Jan 24;18:12. doi: 10.1186/s12887-017-0977-7 (PMC5784678; doi:10.1186/s12887-017-0977-7)
Supplement: Supplementary file 2 — The how and why of parents’ formula feeding practices – further supporting quotes. (DOCX 21 kb) [file 12887_2017_977_MOESM2_ESM.docx]

Appendix 2. The how and why of parents’ formula feeding practices

| **Aspect of infant feeding** | **Theme** | **Supporting quotes** |
| --- | --- | --- |
| **Choice of formula** | **What’s on (and in) the tin** | Relying on information derived from marketing:  *We just watched ads on TV and they just told us what - well the ads say what the formula's about like what it does to their brains and their digestive system so that's how we chose to put her on [brand].* Hayley, switched early  *…they all claim to be you know, “do this for your baby’s brain” and “this for their body”, and you wonder is it really, is the other tin that doesn’t have that on the label really not going to do those things? It’s really hard to know if you’re doing the right thing.* Lucy, formula fed  Requested more input from health professionals:  *I think at the hospital it would be good if they could give you information on all the different stuff that’s out there, not necessarily pointing you towards one or another but just what’s in them, how they can benefit the child, whether the products are imported, that kind of stuff because that’s fairly important. You want to know where it’s coming from.* Alyssa, switched early  *Yeah, it would’ve been really good to have* (information about the ingredients in formula) *because then I could’ve been more aware of what was in it and the benefits kind of thing of it… So if you had that information out there, then yeah, for sure.* Amelia, formula fed  *A little bit more information about which ones work best for the situation. Some babies I know go onto formula fairly early so it’d be great if we could have some information about why certain formulas are better for certain situations.* Chloe, switched late |
| **Bottle preparation** | **Mostly by the tin** | Information regarding formula preparation was obtained predominantly from the manufacturers’ advice:  *…I always made sure that the water and scoop ratio was correct and that the scoop was completely level as per the instructions.* Evelyn, mix fed  *Generally, no. I mean I never really had fully compacted scoops, it was always loosely packed. So there could be that possibility there might be a little less or more in the scoop but generally speaking I would never add…Say for instance on the tin it says 60ml of water: one scoop, I would never add one and a half scoops or two or any less but like I said there was that give or take.* Amelia, formula fed  *No I don’t - I know that if you make it up differently it can make them sick so I kind of make it up exactly the amount it says except she’s having different amount for her age group, I make it up as it’s recommended.* Isla, switched early |
| **How much and how often** | **Demand versus routine** | Organic routine determined by infant perceived needs which parents perceived had a pattern:  *She was in a pretty good routine from about six weeks old. So yeah, basically she’d let me know when she was hungry, the majority of the time either before or after a sleep, depending on how she’d gone through the night because she slept right through from six weeks old.* Alyssa, switched early  Sleep and daily routine influence:  *…it would depend if we had something on where we were out, it would depend on whether there was facilities for me to feed or to top him up with the formula.* Emily, mix fed  *Just by what was happening that day I guess, like if we were going out I didn’t feel comfortable feeding her in public so I would take a bottle of express milk or if I wasn’t going to be around…she’d have express milk but if I was at home she was usually on the boob.* Isla, switched early  *She enjoys the bottle when she’s in the car. So I try and sometimes go out and get my errands while she’s got a bottle in the car seats. That’s in the mornings.* Sienna, mix fed  Time between feeds:  *So you're supposed to feed your baby every three to four hours so as soon as they wake up for the day they have a feed and then every three to four hours from there you give them another feed and so on so then you just work out which is the best routine.* Charlotte, switched late  *I kind of, used the feeding guide and if it said five or six feeds based on his age, I’d kind of work out roughly if you took 24 hours in a day and evened that out, roughly how many hours between feeds would that be. Then used that along with my signs, the signs of [baby] when he was hungry to match roughly those times up as closely as possible. If he was hungry about that time great, especially in the early days you try and feed them every four hours or whatever it is that’s recommended.* Evelyn, mix fed  *When I first went into hospital because I was breastfeeding the nurses said when you breastfeed it's usually every two hours and with formula it's four hours so we went roughly on that. Then my partner's mum said "usually babies only wake up when they're hungry" so we also used that as a help as well.* Hayley, switched early |
|  | **Balancing responding to baby’s cues versus the information on the tin** | Trial and error:  *I slowly kind of realised that was her hungry sign and I would try it some days. It was sometimes a fail thing, like I’d try her and she just wasn’t hungry and there was some other reason. But I’d always give it a go.* Amelia, formula fed  *…we just kind of guessed. If she was crying we'd cuddle her or we'd feed her, change, cuddle. If she was still crying we'd just have to keep holding her and playing with her and distracting her.* Hayley, switched early  *I went by feeling because I sort of knew when she was hungry or when she wanted a bottle or wanted to feed. So it was me reading my own child really. So yeah, it was a gradual process and also going by the instructions on the tin of how much formula that child should be having at that age.* Sienna, mix fed  *Well, I think the guide on the tin is pretty good and then you’ve just really got to listen to your baby and watch your baby’s body language as to whether they actually want it or not, you know, it’s trial and error. If you’re in tune with your baby, you’ll be right.* Alyssa, switched early  Always made up tin amount even if baby did not always drink it:  *Well I'm making up 180 mils with six scoops but she's not actually drinking quite all of it but I still make it to that level just so if she happens to want the little bit extra.* Emma, switched early  *I feel like I'm giving mine too much at the moment. But I'd rather put more in there than less... I'm doing about 180 mils at the moment but I probably feel that she probably only needs about 120 but it's different each feed.* Imogen, switched late  *I went for the normal portion you’d give her. Yeah. So if she finished it, she finished it, if she didn’t, because I would never be able to overfeed her because she would always turn away when she’s had enough.* Sienna, mix fed |
|  | **Perceptions of other external cues** | Offer the bottle again  *She just pushes it away and I try and offer it again just in case but nine times out of 10 she’ll push it away and not be bothered with it anymore.* Ellie, switched early  *Well if she said no the first time I'd offer it again and if she said no the second time I'd sit her up and give her a burp and give her a two/three minute break and then give it to back to her. Then after that time if she did it again well that means she's finished.* Hayley, switched early  Finished bottle indicating appetite:  *If he … did start whinging again, you would offer the bottle again but generally, he was fine. But he came pretty close to finishing it most times. So I knew that he had a decent amount.* Abigail, formula fed  *So we sort of looked at the tin to see what they thought and went from there to see how much he actually did drink from their recommendation and then make it up to that from then on until he was actually finishing a bottle and you know, still looking for more as he got bigger.* Lucy, formula fed |
|  | **Getting advice** | *Initially it was I just followed the guideline of the 150 mils per their weight, and then I kind of just let it… I just asked other mums and how much they feed at a certain age.* Zara, formula fed  *Just, you know, if the baby sort of wasn’t taking a full bottle… So it was just the experience other mums had been through saying “Well, you know, they’re obviously getting enough, they know when they’ve had enough and they don’t over indulge on it”, so...* Emily, mix fed  *But then the tin tells you that they should be drinking 180ml. So I actually ended up taking her – because I was freaking out that she wasn’t getting enough and, you know, people around you saying, “Oh, she’s not drinking enough” and all the rest of it. I actually took her to the health clinic and had her weighed and all the rest of it and the midwife there said, “You know what? Don’t listen to anyone else. You know your baby and you know what she’ll take.” After that advice, pretty much I’d make up the additional whatever the tin was telling me and it would take her about a week, maybe a week and a half to take that on but otherwise she was – yeah.* Alyssa, switched early |
