# Supplementary material for: Infant formula feeding practices and the role of advice and support: an exploratory qualitative study
Source: BMC Pediatr. 2018 Jan 24;18:12. doi: 10.1186/s12887-017-0977-7 (PMC5784678; doi:10.1186/s12887-017-0977-7)
Supplement: Supplementary file 3 — Sources of information, advice and support about formula and social environment – further supporting quotes. (DOCX 19 kb) [file 12887_2017_977_MOESM3_ESM.docx]

Appendix 3. Sources of information, advice and support about formula and social environment

| **Sources of information, advice and support about formula** | **Informal sources of advice** | Informal sources:  *I would probably say word of mouth from friends would influence me the most, rather than say advertising or medical professionals.* Ellie, switched early  *I think it’s the way formula is made and also how the back of the tin is very clear, I feel that’s great and also you’re quite supported from other mothers… In terms of professional advice, probably not in some ways. I just feel like the maternal and child health nurse could step up a little bit maybe. But overall we feel we have been supported.* Chloe, switched late  The internet:  *If I had any question I generally just went online and then just…if it was my forum so it was like other mums had written in with other mums writing back to them.* Emily, mix fed  *I did a lot of Googling on how to use it, the best times to give it to her…yeah, they didn’t, I mean my maternal health nurse didn’t really offer that much advice when it came to formula, it was probably more is she drinking enough.* Amelia, formula fed  Felt satisfied with available sources:  *…the tin's pretty good - because it even has pictures on the side on how to sanitise bottles and how to use the correct weight for the formula to scoop and how much water to put in.* Kim, formula fed  *Well I don't think we have really needed any extra advice. If we do need it we can just Google it or go see our nurse or the normal GP.* Hayley, switched early  *Apart from the tin and the lactation consultant and the maternal health nurses, I haven’t found that there’s much more out there. I mean, you get advice from fellow parents but that’s – yeah […would you have liked more?] No, I think it was sufficient.* Zoe, mix fed  *I think the information on the tin pretty much tells you how to do it all, like how to measure it out and how much to give and all that kind of stuff. So I don’t – like, for me personally I don’t feel like I would have benefitted from having some extra information for formula. Yeah.* Savannah, mix fed  Formula is easy:  *Yeah, no the formula feeding is fine. Its feeding solids that have been difficult I think.* Lucy, formula fed  *…yeah no problems with formula. She took to a bottle like glue which is good… But the formula and milk, the mix, she’s just, yep, she’s into it.* Sienna, mix fed |
| --- | --- | --- |
|  | **Formal sources of advice** | Timing:  *It's interesting sort of stepping back and going gosh I probably should have done a bit more research or I probably should have - but I think sometimes there's not a lot of time when it comes to formula. You kind of need to get on it pretty quickly.* Imogen, switched late  *I did have some discussions again with the… the girl from mothers group and I, because the main reason I went to formula was because I was going back to work. So I had a sort of limited time to get her onto formula…* Chloe, switched late  *The first brand I got made him severely constipated. So I went to the pharmacist and it was only a Sunday so it was hard to get into a doctor to talk about it and they recommended the brand that we’re using because it was gentle on tummies and we’ve had no problems since.* Lucy, formula fed  *So I sent my partner to go into the supermarket and get it and then he just picked up the one that said from birth that he saw straight away so that's how we picked it.* Kim, formula fed  Health professionals don’t talk about it:  *…it was quite hard to get any solid information, there was no documentation or anything like say whenever I went and visited the doctors or went and got her check-up… they’d often ask me “Am I breastfeeding or formula feeding?” When I said I was formula feeding they’d just document it down, they wouldn’t actually talk about.* Ellie, switched early  *…they just said that they weren't allowed to say use this formula because that's what we use because they don't have any affiliation with them so we just went out on the wing.* Kim, formula fed  Breastfeeding agenda:  *Just be more accepting of the fact that I was formula feeding and not try and force you to change your mind to breastfeed and I kind of had to search for the information… So I had to always seek out the information anything to do with formula feeding to prepare myself… Because I never formula fed from birth before. I always tried breastfeeding. So, that part of it was all new and they didn’t really offer a lot of information.* Abigail, formula fed  *I guess I’ve been jaded a little bit in terms of professional advice just from that one episode that I told you [previously this participant said ‘…went to the maternal and child health nurse to ask them how I go about introducing formula, from going from breastfeeding to formula feeding. I was met with a very negative attitude and was told…basically I wasn’t given any advice at all, just told that I should continue breastfeeding because it was the best thing for my baby’]… So that kind of jaded me then in terms of going and seeking other professional help, I just went “Oh well, I’ll just work it out myself.”* Chloe, switched late  Comfortable but proactive in going to health professionals:  *I kind of got the information from her [postnatal home visiting nurse] and same thing happened second time and third child I knew I was just going to formula feed. I would ask, they don’t generally volunteer formula information unless you ask.* Abigail, formula fed  *I think you can get information; you’ve just got to go and source it. You’ve just got to go to the clinics and talk to people and stuff. The information is there it's just not handed out.* Zara, formula fed  *Going to the professionals, I found it really quite easy and it’s always been quite helpful.* Lucy, formula fed |
| **Social environment** | **Bottle stigma** | Bottle stigma and feeling judged:  *I think formula is one of those second rate things when it comes to the community, not to say that you get frowned upon but if you do choose to go formula a lot of people are like, “Oh, okay you’re not breastfeeding?”, you know - and there’s that stigma to it.* Amelia, formula fed  *I think there's still that stigma out there a lot…why are you using formula, you should be breastfeeding blah, blah, blah, blah.* Imogen, switched late  *You get a bit frowned upon a bit. Like as in the general public…They're kind of just like “oh you're a formula fed baby, why did you formula feed for” kind of look they kind of give.* Kim, formula fed  *…with my son I dumped them* [mother’s group] *because they were all ‘breast is best’ and stuff like this and then…I met up with them a few months later…and they all stopped breastfeeding. And I’m thinking, you’re the people who made me feel so guilty…* *And I’m there going – “you were judging me? Okay…”* Layla, switched early  Formula feeding as norm for some communities and families:  *Friends and family mostly, a lot of them because they’d all formula fed themselves…they weren’t necessarily negative towards breastfeeding, but they didn’t understand the challenges of it.* Ellie, switched early  *Probably I would have liked more support when breastfeeding from my family but I didn't get much support.* Hayley, switched early |
